# Evidence for a neuroprotective microRNA pathway in amnestic mild cognitive impairment

**DOI:** 10.3389/fnins.2015.00430

**Published:** 2015-11-05

**Authors:** Rebecca B. Weinberg, Elliott J. Mufson, Scott E. Counts

**Affiliations:** ^1^Department of Translational Science and Molecular Medicine, Michigan State UniversityGrand Rapids, MI, USA; ^2^Department of Neurobiology, Barrow Neurological InstitutePhoenix, AZ, USA; ^3^Department of Family Medicine, Michigan State UniversityGrand Rapids, MI, USA; ^4^Hauenstein Neuroscience Center, Mercy Health Saint Mary's HospitalGrand Rapids, MI, USA

**Keywords:** Alzheimer's disease, mild cognitive impairment, frontal cortex, microRNAs, sirtuin 1, miR-23a, miR-212, gene expression regulation

## Abstract

MicroRNAs (miRNAs) that regulate mRNA stability have been linked to amyloid production, tau phosphorylation, and inflammation in Alzheimer's disease (AD). However, whether cerebral miRNA networks are dysregulated during the earliest stages of AD remains underexplored. We performed miRNA expression analysis using frontal cortex tissue harvested from subjects who died with a clinical diagnosis of no cognitive impairment (NCI), amnestic mild cognitive impairment (aMCI, a putative prodromal AD stage), or mild AD. Analysis revealed that the miRNA clusters miR-212/132 and miR-23a/23b were down-regulated in the frontal cortex of aMCI subjects. Both miR-212/132 and miR23a/b are predicted to destabilize the message for sirtuin 1 (sirt1); hence, down-regulation of either miR-212/132 or miR-23a/b in frontal cortex should promote sirt1 mRNA expression in this region. qPCR studies revealed that frontal cortex levels of sirt1 were increased in aMCI. Given the ability of frontal cortex to respond to the onset of dementia by neuronal reorganization, these data suggest that miRNA-mediated up-regulation of the sirt1 pathway represents a compensatory response to the onset of the disease. By contrast, qPCR analysis of inferior temporal cortex, an area affected early in the progression of AD, showed no changes in miR-212/132, miR-23a/b, or sirt1 transcripts in the same aMCI subjects. *In vitro* mechanistic studies showed that coordinated down-regulation of miR-212 and miR-23a increased sirt1 protein expression and provided neuroprotection from β-amyloid toxicity in human neuronal cells. Taken together, these data suggest a novel miRNA-mediated neuroprotective pathway activated during the progression of AD that may be amenable to therapeutic manipulation.

## Introduction

Progress in slowing the course of Alzheimer's disease (AD) has been confounded by a lack of disease modifying therapeutics. Given the vast complexity of this multisystem disorder, therapeutic development will likely depend on a deeper understanding of the intricate molecular mechanisms that regulate the maintenance and survival of selectively vulnerable neuronal populations during disease progression. In this regard, the presence of small non-coding microRNAs (miRNAs) that negatively regulate mRNA stability (Lagos-Quintana et al., 2001) presents an underexplored mechanism for fine-tuning gene expression within complex cellular networks, which likely plays a pivotal role in the balance between health and disease (Nelson et al., 2008; Hébert and De Strooper, 2009). Select miRNAs regulate diverse brain functions including neurogenesis and differentiation, synaptic plasticity, and energy metabolism (Schratt et al., 2006; Aschrafi et al., 2008; Fineberg et al., 2009; Rajasethupathy et al., 2009). This widespread influence of miRNA regulation on neuronal physiology suggests that perturbations in miRNA function could be involved in the pathogenesis of complex neurodegenerative disorders including AD (Nelson et al., 2008; Hébert and De Strooper, 2009). Indeed, AD brains display altered expression of several miRNAs that regulate β-secretase BACE1, a key enzyme involved in the generation of amyloid-β (Aβ) plaque pathology (Hébert et al., 2008; Wang et al., 2008). In addition, miRNA dysregulation has been linked to tau phosphorylation (Hébert et al., 2010; Absalon et al., 2013; Banzhaf-Strathmann et al., 2014) and pro-inflammatory activity (Cui et al., 2010; Lukiw et al., 2010; Li et al., 2012). However, whether miRNA networks are dysregulated in the brains of people in the putative prodromal stages of AD such as amnestic mild cognitive impairment (aMCI) (Yaffe et al., 2006; Albert et al., 2011) and the extent to which these changes have physiologic consequences for the onset of AD remain unclear. To begin to address these knowledge gaps, we performed microarray and quantitative PCR (qPCR) studies to compare the levels of miRNAs isolated from frontal cortex (Brodmann area 10) and inferior temporal cortex (Brodmann area 20) tissue obtained postmortem from people who died with a clinical diagnosis of no cognitive impairment (NCI), aMCI, or AD. We report that two families of miRNAs, miR-212/132 and miR-23a/b, were down-regulated in frontal cortex in aMCI and AD compared to NCI, yet remained stable in inferior temporal cortex. Down-regulation of either miRNA family was predicted to up-regulate the deacetylase sirtuin 1 (sirt1), which is involved in mediating protective neuronal cell stress responses (Brunet et al., 2004; Qin et al., 2006; Bonda et al., 2011). Sirt1 mRNA levels were higher in frontal cortex of aMCI subjects but stable in inferior temporal cortex, suggesting a link between miR-212/132 and miR-23a/b down-regulation and reduced transcriptional repression of sirt1 target mRNA. Experimental down-regulation of miR-212 and miR-23a in cultured neurons up-regulated sirt1 and provided neuroprotection against Aβ toxicity. Given the relatively delayed involvement of frontal cortex in AD pathogenesis and the ability of this region to respond to the onset of dementia by neuronal reorganization (DeKosky et al., 2002; Counts et al., 2006; Bell et al., 2007; Williams et al., 2009; Bossers et al., 2010), these data suggest that miRNA-mediated up-regulation of sirt1 is a novel neuroprotective pathway activated during prodromal AD.

## Materials and methods

### Subjects

This study was exempt from IRB approval following guidelines for using de-identified postmortem tissue administrated by the Rush University Medical Center. Frontal and inferior temporal cortex tissue was obtained postmortem from 32 participants in the Rush Religious Orders Study (Bennett et al., 2002) who were clinically diagnosed within a year of death with NCI (*n* = 12), aMCI (*n* = 10), or AD (*n* = 10; see Table 1). Details of clinical evaluations and diagnostic criteria have been previously published (Mufson et al., 1999; Counts et al., 2006; Ginsberg et al., 2010). In addition to an annual clinical evaluation, subjects were administered the Mini Mental State Exam (MMSE) and a battery of 19 neuropsychological tests referable to multiple cognitive domains (e.g., episodic memory, perceptual speed, Mufson et al., 1999). A Global Cognitive Score (GCS), consisting of a composite z-score calculated from this test battery, was determined for each participant (Bennett et al., 2002). The MCI population was defined as subjects who exhibited cognitive impairment on neuropsychological testing but who did not meet the clinical criteria for AD recommended by the joint working group of the National Institute of Neurologic and Communicative Disorders and Stroke/AD and Related Disorders Association (NINCDS/ADRDA) (McKhann et al., 1984; Bennett et al., 2002). The aMCI diagnosis is based on impairments in episodic memory (Yaffe et al., 2006; Albert et al., 2011). These criteria are compatible with those used by experts in the field to describe subjects who are not cognitively normal but do not meet established criteria for dementia (Petersen et al., 2001). “Other dementia” (OD) neurologic controls (*n* = 5) used in the analysis included three multi-infarct dementia and two Lewy body dementia cases.

**Table 1:** **Clinical, demographic, and neuropathological characteristics by diagnosis category**.

	**Clinical Diagnosis**				
	**NCI (*N* = 12)**	**aMCI (*N* = 10)**	**AD (*N* = 10)**	***P*-value**	**Pair-wise comparison**
Age (years) at death: Mean ± SD (Range)	83.0 ± 5.9 (67–92)	82.9 ± 4.9 (72–90)	88.6 ± 7.0 (76–97)	0.3^a^	–
Number (%) of males:	6 (50%)	5 (50%)	4 (40%)	0.4^b^	–
Years of education: Mean ± SD (Range)	16.8 ± 3.4 (12–20)	18.8 ± 2.7 (12–22)	18.2 ± 3.4 (15–23)	1.0^a^	–
Number (%) with ApoE ε4 allele: MMSE: Mean ± SD (Range)	0	3 (33%)	4 (40%)	0.007^b^	NCI < AD
	28.3 ± 1.7 (26–30)	28.0 ± 1.3 (26–30)	17.5 ± 8.1 (7–28)	< 0.001^a^	(NCI, MCI) > AD
Global cognitive score: Mean ± SD (Range)	0.06 ± 0.3 (−0.3–0.7)	−0.03±0.4 (−0.6–0.2)	−1.3±0.8 (−2.5–0.1)	< 0.0001^a^	(NCI, MCI) > AD
Postmortem interval (hours): Mean ± SD (Range)	5.7 ± 2.4 (3.0–12.4)	5.8 ± 3.1 (2.0–10.6)	4.8 ± 3.2 (1.5–12.3)	0.5^a^	–
Distribution of Braak scores: 0 I/II III/IV V/VI	0 5 7 0	0 4 5 1	0 1 3 6	< 0.001^a^	(NCI, MCI) < AD
NIA Reagan diagnosis: No AD Low Intermediate High	0 7 3 0	0 5 5 0	0 1 4 5	< 0.001^a^	(NCI, MCI) < AD
CERAD diagnosis: No AD Possible Probable Definite	3 2 4 1	4 3 3 0	0 0 5 5	0.008^a^	(NCI, MCI) < AD

a *Kruskal-Wallis test, with Bonferroni correction for multiple comparisons*.

b *Fisher's exact test, with Bonferroni correction for multiple comparisons*.

Tissue samples were accrued as previously reported (Mufson et al., 1999; Counts et al., 2006; Ginsberg et al., 2010). At autopsy, tissue from one hemisphere was immersion-fixed in 4% paraformaldehyde in 0.1 M phosphate buffer, pH 7.2 for 24–72 h at 4°C. Tissue slabs from the opposite hemisphere were frozen at −80°C prior to biochemical analysis. Series of fixed tissue sections were prepared for neuropathological evaluation including visualization and quantitation of neocortical and hippocampal amyloid plaques and neurofibrillary tangles (NFTs) using antibodies directed against Aβ peptide (Aβ; 4 G8, Covance), tau (PHF1, a gift from Dr. Peter Davies) (Mufson et al., 1999; Bennett et al., 2002) as well as thioflavine-S histochemistry and a modified Bielschowsky silver stain. Additional sections were stained for Lewy bodies using antibodies directed against ubiquitin and α-synuclein. Exclusion criteria included argyrophilic grain disease, frontotemporal dementia, Lewy body disease, mixed dementias, Parkinson's disease, and stroke. A board certified neuropathologist blinded to the clinical diagnosis performed the neuropathological evaluation. Neuropathological criteria were based on NIA-Reagan, CERAD, and Braak staging (Braak and Braak, 1991; Mirra et al., 1991; Hyman et al., 2012). Amyloid burden and apolipoprotein E (ApoE) genotype were determined for each case as described previously (Mufson et al., 1999; Bennett et al., 2002).

### miRNA expression profiling

A pilot miRNA microarray screen (Exiqon miRCURY LNA microarray v.11, ~1360 array features including synthetic spike-in miRNA controls) was performed using RNA derived from postmortem frozen frontal cortex tissue from three NCI [age = 86.7 ± 1.5 (mean ± *S.D*.) years, MMSE = 29.7 ± 0.6, PMI = 5.2 ± 1.5 h] and three AD (age = 87.7 ± 1.5, MMSE = 21.7 ± 2.5, PMI = 5.2 ± 1.7) subjects. Approximately 30% of the miRNAs on the array were proprietary and not available for analysis in public databases. Based upon the microarray pilot data, we performed qPCR validation for select transcripts using frozen frontal cortex and temporal cortex from each of the NCI, aMCI, AD, and OD neurologic control cases collected for the full study. Total RNA was extracted (miRvana, Ambion) and RNA integrity and concentration was verified using Bioanalysis (Agilent). Samples were assayed on a real-time PCR cycler (7900HT, Applied Biosystems) in 96-well optical plates as described previously (Counts et al., 2007; Ginsberg, 2008; Alldred et al., 2009). Target miRNAs of interest as well as the RNU48 artificial normalization control were amplified using specific Taqman hydrolysis probe sets (Applied Biosystems). In addition, Taqman probe sets specific for sirt1 and control glyceraldehyde 3-phosphate dehydrogenase were used to quantify sirt1 transcript levels in the same samples. The ddCT method was employed to determine relative expression levels of each amplicon (Counts et al., 2007; Ginsberg, 2008; Alldred et al., 2009). Variance component analyses revealed relatively low levels of within-case variability, and the average value of the triplicate qPCR products from each case was used in subsequent analyses.

### Dual *in situ* hybridization/immunohistochemical localization of miR-23a and sirt1

*In situ* hybridization to detect miR-23a was performed on 10 μm, cryostat-sectioned samples of frozen frontal cortex using a digoxin (DIG)-labeled hsa-miR-23a probe (Exiqon), adapting the protocol of Doné and Beltcheva (2014). Briefly, tissue sections were fixed in 10% neutral buffered formalin overnight at room temperature (RT). The next day, sections were treated with 20 μg/mL proteinase K for 10 min at 37°C followed by hybridization with 400 nmol hsa-miR-23a probe for 1 h at 55°C. The sections were then blocked with 2% sheep serum/1% bovine serum albumin for 15 min at RT followed by incubation with alkaline phosphatase-conjugated sheep anti-DIG Fab fragments (1:500, Roche) for 1 h at RT. The sections were then incubated with the alkaline phosphatase substrates NBT (nitro blue tetrazolium)/BCIP (5-bromo-4-chloro-3-indolyl-phosphate; Roche) for 2 h at 30°C revealing a dark purple reaction product. Following miR-23a visualization, the sections were incubated overnight at 4°C with a rabbit anti-sirt1 monoclonal antibody (1:100, Origene) in Tris-buffered saline (TBS, pH 7.4)/0.25% Triton X-100/1% normal goat serum. Following TBS rinses, the sections were incubated with horseradish peroxidase-conjugated goat anti-rabbit secondary antiserum (Vector Laboratories) for 1 h at RT. Sirt1 labeling was accomplished by serial incubations in ABC peroxidase reagent (Vector Laboratories) and 3, 3′diaminobenzidine tetrahydrochloride hydrate at RT to reveal a brown reaction product.

### Neuronal cell culture

hNT neuronal cultures were derived from the human teratocarcinoma NT2 cell line (a gift from Dr. Virginia Lee, Univ. Penn) (Andrews et al., 1984; Counts and Mufson, 2010). NT2 cells were maintained in OptiMem (Invitrogen) with 5% fetal bovine serum (FBS). For differentiation, cells were seeded at 25,000/cm^2^ into T75 flasks in 1:1 DMEM/F-12 media (Invitrogen)/10% FBS, treated twice a week with 10 μM all-*trans* retinoic acid (Sigma) for 4 weeks and then seeded to new T75 flasks at 650,000/cm^2^ and treated with the mitotic inhibitors cytosine arabinoside (1 μM) and fluorodeoxyuridine (10 μM, Sigma) for 2 weeks. This resulted in a layer of phase-bright, post-mitotic neuronal cells loosely attached atop a monolayer of non-neuronal cells. Neuronal enrichment was achieved by gently trypsinizing the top neuronal layer and replating at 125,000/cm^2^ onto 2% Matrigel (BD Biosciences) and 10 μM poly-D-lysine (Sigma)-coated black-walled 96 well plates (cell viability) or 60 mm dishes (western blotting) (BD Biosciences) in 1:1 DMEM/F-12 media/10% FBS (Counts and Mufson, 2010).

### miRNA inhibition and functional validation

hNT cultures were transfected with small miRNA inhibitors (miRCURY LNA inhibitors, Exiqon) specific for miR-212, miR-132, miR-23a, miR-23b, or an inhibitor negative control sequence (Exiqon) (*n* = 8/treatment group in three independent experiments). hNT neurons were plated at 20K/cm^2^ and incubated with 50 nM inhibitor/1% Lipofectamine RNAiMAX (Life Technologies) in OptiMem for 18 h, then exchanged into 1:1 DMEM/F-12 media/10% FBS for 36 h prior to experimentation.

### Quantitative western blotting

hNT neurons were harvested 36 h post-transfection and separated into nuclear and cytosolic fractions (NE-PER, Pierce) for quantitative immunoblotting. Nuclear proteins were solubilized in loading buffer and separated by SDS-PAGE, transferred to Immobilon-P membranes (Millipore), blocked in Tris buffered saline (pH 7.4) containing 0.1% Tween-20 and 2% nonfat milk, and then incubated overnight at 4°C with rabbit polyclonal antiserum to sirt1 (1:500; Chemicon) and a mouse monoclonal antibody to lamin A (1:500, Abcam) as a loading control for the nuclear fraction. Blots were then incubated for 1 h with horseradish peroxidase-conjugated goat anti-rabbit (Bio-Rad; 1:5000) and anti-mouse (1:8000; Pierce, IL) IgG secondary antibodies and reactivity was quantified using Kodak 1D image analysis software (Perkin-Elmer). Each sample was analyzed on three different Western blots in independent experiments. Signals for sirt1 were normalized to lamin A for quantitative analysis (Counts et al., 2004, 2006).

### Amyloid toxicity experiments

Following miRNA inhibitor or control transfections, differentiated hNT neurons were challenged with 10 μM Aβ_1−42_ for 48 h in the presence or absence of the sirt1-specific inhibitor EX527 (100 nM, Tocris). Aβ_1−42_ was dissolved in DMSO and pre-aggregated for 16 h at 37°C. Western blotting revealed an accumulation of SDS-soluble immunoreactive material migrating at ~40–48 kDa reminiscent of oligomeric amyloid (Walsh et al., 1999). Neuronal viability was determined by propidium iodide (PI) retention (Counts and Mufson, 2010).

### Statistical analysis

miRNA levels quantified by qPCR were compared among the NCI, aMCI, AD, and OD neurologic controls via One-way ANOVA with Bonferroni *post-hoc* testing. The relationship between specific miRNA and mRNA levels was assessed by Spearman rank correlations. Quantitative Western blotting data of control and miR-treated samples were analyzed by unpaired *t*-tests. Finally, cell death comparisons in the different cell culture treatment groups were assessed by One-way ANOVA with Bonferroni *post-hoc* testing. The level of statistical significance was set at α = 0.05 (two-tailed).

## Results

### Subject demographics

The clinical diagnostic groups did not differ by age, gender, years of education, or postmortem interval (Table 1). There were more subjects with an ApoE 4 allele in the aMCI (33%) and AD (44%) groups than in the NCI group (0%). AD cases had significantly lower MMSE scores compared to both aMCI and NCI cases (*p* < 0.001), whereas the latter two groups did not differ statistically (Table 1). GCS *z*-scores were significantly lower in AD compared to the NCI and aMCI groups (*p* < 0.0001). Subjects in the different clinical diagnostic groups displayed considerable heterogeneity with respect to pathological diagnostic criteria. Neuropathological examination revealed that 58% of NCI, 60% of aMCI, and 90% of AD cases were classified as Braak stages III–VI. Using NIA-Reagan criteria, 25% of NCI, 50% of aMCI, and 90% of AD cases were classified as intermediate to high likelihood of AD (Table 1). For CERAD diagnosis, 42% of NCI, 30% of aMCI, and 100% of AD cases received a diagnosis of probable/definite AD. Statistical analysis revealed that the AD group displayed significantly higher pathology than the NCI and aMCI groups based on Braak (*p* < 0.001), NIA-Reagan (*p* < 0.001) and CERAD (*p* = 0.008) diagnosis.

### miRNA expression dynamics during the progression of AD

**Figure 1:**
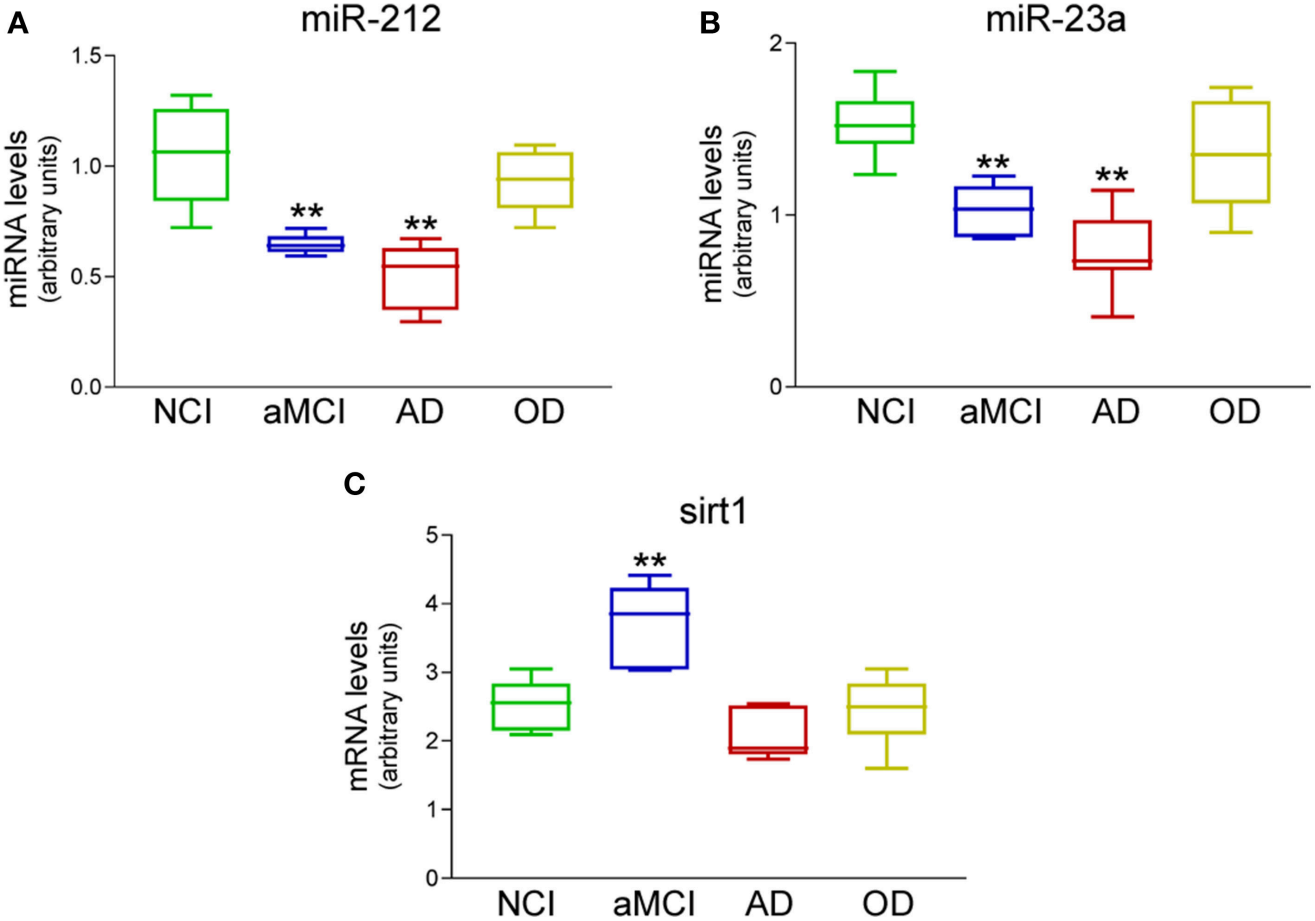
**Differential expression of miR-212, miR-23a, and sirt1 transcripts in the frontal cortex of aMCI subjects**. qPCR analysis was performed on frozen frontal cortex tissue samples harvested from NCI (*n* = 12), aMCI (*n* = 10), mild AD (*n* = 10), and other dementia (OD, *n* = 5) neurologic control subjects. Box plots show that **(A)** miR-212 and **(B)** miR-23a were significantly down-regulated by ~50% in aMCI and by ~60% in AD. **(C)** Their predicted mRNA target sirt1 was up-regulated by ~40% in aMCI. miR expression levels were normalized to the human RNU48 control miRNA, whereas sirt1 mRNA was normalized to GAPDH for quantitative analysis. **p < 0.01 via One-way ANOVA with Bonferroni correction for multiple comparisons.

**Figure 2:**
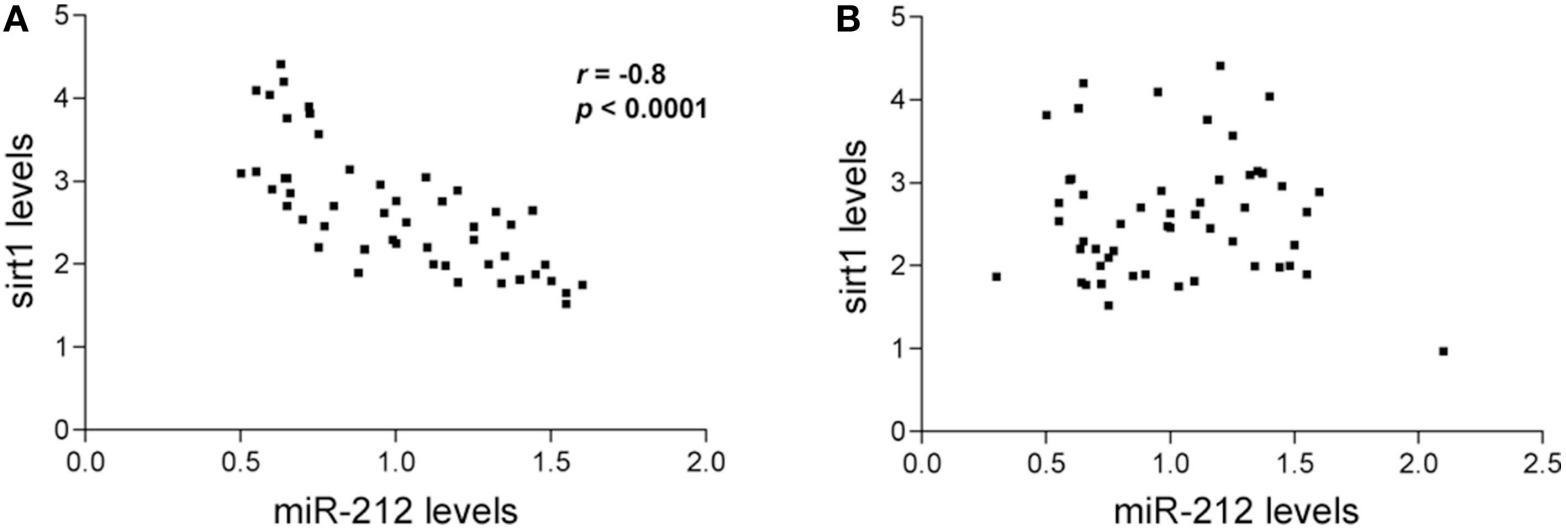
**Inverse relationship between miR-212 and sirt1 expression in the frontal cortex**. Spearman rank correlations were performed to test for relationships between miR-212 and sirt1 expression. Scatterplots show that lower miR-212 transcript expression was associated with higher sirt1 transcript expression in **(A)** frontal cortex (*r* = −0.81, *p* < 0.0001), but not in **(B)** temporal cortex in the subjects examined.

A small pilot miRNA microarray screen performed on RNA isolated from frozen frontal cortex tissue from three NCI and AD cases showed that the expression levels of 30 miRNAs were significantly different between the two groups, along with miRNAs that showed high but non-significant magnitudes of expression change between the two groups. The top 100 miRNAs are listed in Table 2. We performed qPCR to measure select miRNAs in frontal cortex across all groups examined. Specifically, we tested the expression levels of 20 of the top 100 miRNAs based on significant differences, magnitude of expression level difference and/or interest based on a literature search (Cogswell et al., 2008; Hébert et al., 2013; Lau et al., 2013; Wong et al., 2013) (Table 3). Of the 20 candidate miRNAs evaluated, miR-498, and miR-150 were significantly up-regulated in AD frontal cortex; miR-150 was up-regulated in aMCI. Several miRNAs were also significantly down-regulated in AD frontal cortex, including miR-886-3p, miR-132, miR-21, miR-23a, miR-140-3p, miR-212, miR-23b, let-7d, and miR-181a (Table 3). However, two distinct clusters of these miRNAs, miR212/132, and miR 23a/23b, were also significantly down-regulated by ~50% in the frontal cortex of aMCI subjects relative to NCI subjects (Table 3, Figures 1A,B). By contrast, qPCR analysis of temporal cortex revealed that miR-212 and miR-132 expression levels were decreased only in the AD group, whereas miR-23a and miR-23b were unchanged across the clinical diagnostic groups (Table 4). Since miRNAs negatively regulate transcript stability, down-regulation of the miR-212/132 and miR-23a/b clusters in frontal cortex would be predicted to promote the stability of their target mRNAs in aMCI. Given several lines of evidence that the frontal cortex undergoes neuroplastic, presumably neuroprotective remodeling in the face of mounting AD neuropathology during MCI (DeKosky et al., 2002; Counts et al., 2006; Bell et al., 2007; Williams et al., 2009; Bossers et al., 2010), we searched the miRanda and miRbase prediction algorithm databases (microRNA.org) for mRNA targets of these miRNAs that might mediate potentially compensatory pathways to promote neuronal viability. Surprisingly, all four miRNAs were predicted to target sirt1, a histone deacetylase involved in mediating neuronal cell stress responses (Brunet et al., 2004; Qin et al., 2006; Bonda et al., 2011). To determine whether target sirt1 transcripts were also differentially regulated in the frontal and temporal cortex in aMCI, we performed qPCR analysis of sirt1 expression levels in the same tissue samples used for miRNA analysis. This analysis revealed that sirt1 mRNA levels were significantly up-regulated by ~40% in the frontal cortex of aMCI compared to NCI and AD subjects (Figure 1C). By contrast, sirt1 levels were stable in temporal cortex across the diagnostic groups (Table 4). Furthermore, increased sirt1 mRNA expression was significantly associated with decreased miR-212 levels frontal cortex levels, but this association was not found in the temporal cortex (Figure 2).

**Table 2:** **Top 100 miRNAs dysregulated in AD compared to NCI frontal cortex**.

**Annotation**	***P*-value**	**Average**		**AD vs. NCI**	
		**NCI**	**AD**	**ΔLMR**	**Fold change**
hsa-miR-1285	5.92E-04	0.04	−0.21	−0.25	0.84
hsa-miR-1296	2.00E-03	−0.09	−0.33	−0.24	0.85
hsa-miR-668	5.48E-03	−0.06	−0.57	−0.51	0.70
hsa-miR-551b^*^	6.07E-03	−0.45	−0.01	0.44	1.36
hsa-miR-1826	6.49E-03	−0.11	−0.53	−0.42	0.75
hsa-miR-518e^*^	7.48E-03	−0.44	0.15	0.59	1.50
hsa-miR-132	9.07E-03	0.53	−0.22	−0.74	0.60
hsa-miR-525-5p	1.08E-02	−0.09	0.15	0.24	1.18
hsa-miR-135b	1.40E-02	0.45	0.17	−0.29	0.82
hsa-miR-671-5p	2.23E-02	−0.24	0.00	0.24	1.18
hsa-miR-498	2.68E-02	−0.23	0.29	0.51	1.43
hsa-miR-423-3p	2.76E-02	0.05	−0.17	−0.22	0.86
hsa-miR-886-3p	2.95E-02	0.32	−1.62	−1.94	0.26
hsa-miR-518a-5p	3.76E-02	−0.29	0.07	0.36	1.29
hsa-miR-204	3.97E-02	0.41	0.19	−0.22	0.86
hsa-miR-886-5p	4.21E-02	0.02	−1.49	−1.50	0.35
hsa-miR-132^*^	4.25E-02	0.24	−0.67	−0.91	0.53
hsa-miR-129^*^	4.38E-02	0.20	−0.36	−0.56	0.68
hsa-miR-382	4.08E-02	0.16	−0.03	−0.19	0.88
hsa-miR-921	5.35E-02	−0.58	−0.13	0.45	1.36
hsa-miR-140-5p	5.67E-02	0.33	−0.18	−0.51	0.70
hsa-miR-330-5p	5.87E-02	0.38	0.23	−0.15	0.90
hsa-miR-510	6.01E-02	−0.17	0.12	0.28	1.22
hsa-miR-491-3p	6.15E-02	0.23	−0.35	−0.57	0.67
hsa-miR-320a	7.03E-02	0.29	−0.27	−0.56	0.68
hsa-miR-877	7.05E-02	−0.11	0.17	0.28	1.22
hsa-miR-320b	7.07E-02	0.29	−0.26	−0.55	0.68
hsa-miR-1252	7.13E-02	−0.43	0.10	0.53	1.44
hsa-miR-30e	7.40E-02	0.24	−0.31	−0.55	0.68
hsa-miR-129-3p	7.40E-02	0.21	−0.45	−0.65	0.64
hsa-miR-149	7.43E-02	0.14	−0.26	−0.40	0.76
hsa-miR-25^*^	7.49E-02	−0.21	0.15	0.36	1.28
hsa-miR-149^*^	7.57E-02	−0.02	0.36	0.38	1.30
hsa-miR-483-5p	7.63E-02	−0.29	0.18	0.47	1.39
hsa-miR-630	7.94E-02	−0.16	0.30	0.46	1.38
hsa-miR-1273	8.07E-02	−0.45	0.14	0.59	1.50
hsa-miR-1264	8.34E-02	0.20	−0.12	−0.32	0.80
hsa-let-7a^*^	8.46E-02	0.21	−0.10	−0.31	0.81
hsa-miR-505^*^	8.53E-02	−0.20	0.27	0.47	1.38
hsa-miR-377^*^	8.62E-02	0.00	0.15	0.15	1.11
hsa-miR-21	8.77E-02	0.39	−0.69	−1.08	0.47
hsa-miR-126	8.81E-02	0.14	−0.22	−0.36	0.78
hsa-miR-625	9.22E-02	−0.20	0.11	0.31	1.24
hsa-miR-320d	9.33E-02	0.17	−0.21	−0.39	0.77
hsa-miR-29b-2^*^	9.40E-02	−0.01	−0.33	−0.33	0.80
hsa-miR-29c^*^	9.41E-02	0.13	−0.15	−0.27	0.83
hsa-miR-30a	9.58E-02	0.14	−0.28	−0.41	0.75
hsa-miR-1265	9.70E-02	0.20	−0.03	−0.23	0.85
hsa-miR-135a	1.00E-01	0.51	−0.04	−0.54	0.69
hsa-let-7i	1.04E-01	0.15	−0.33	−0.48	0.72
hsa-miR-589	1.04E-01	−0.14	−0.46	−0.32	0.80
hsa-miR-1201	1.06E-01	−0.20	−0.57	−0.37	0.78
hsa-miR-1259	1.06E-01	0.12	−0.22	−0.34	0.79
hsa-miR-720	1.07E-01	0.18	−0.24	−0.42	0.75
hsa-miR-34b	1.09E-01	0.10	−0.21	−0.32	0.80
hsa-let-7c	1.09E-01	0.19	−0.27	−0.46	0.73
hsa-miR-320c	1.09E-01	0.23	−0.20	−0.43	0.74
hsa-miR-583	1.12E-01	−0.35	0.10	0.46	1.37
hsa-miR-206	1.12E-01	−0.07	0.25	0.32	1.24
hsa-miR-23a	1.12E-01	0.03	−0.44	−0.47	0.62
hsa-miR-181c	1.12E-01	−0.08	−0.44	−0.36	0.78
hsa-miR-769-5p	1.16E-01	0.11	−0.30	−0.41	0.75
hsa-miR-181b	1.17E-01	0.47	−0.46	−0.93	0.53
hsa-miR-1827	1.19E-01	−0.26	0.29	0.55	1.46
hsa-miR-1253	1.20E-01	−0.58	−0.32	0.26	1.19
hsa-miR-301a	1.20E-01	0.16	−0.38	−0.54	0.69
hsa-miR-184	1.21E-01	−0.17	0.15	0.32	1.25
hsa-miR-140-3p	1.21E-01	0.14	−0.46	−0.61	0.66
hsa-miR-33a	1.21E-01	0.31	−0.27	−0.58	0.67
hsa-miR-485-3p	1.22E-01	−0.18	0.16	0.34	1.26
hsa-miR-1274b	1.22E-01	0.21	−0.36	−0.56	0.68
hsa-miR-181a^*^	1.22E-01	0.07	−0.46	−0.53	0.69
hsa-miR-516a-5p	1.23E-01	−0.42	0.02	0.44	1.36
hsa-miR-30d	1.24E-01	0.24	−0.11	−0.35	0.78
hsa-miR-150	1.25E-01	−0.28	0.38	0.66	1.58
hsa-miR-874	1.25E-01	−0.03	−0.24	−0.20	0.87
hsa-miR-339-5p	1.27E-01	−0.03	−0.48	−0.45	0.73
hsa-miR-146b-5p	1.28E-01	0.29	−0.26	−0.55	0.68
hsa-let-7g	1.28E-01	0.10	−0.41	−0.50	0.70
hsa-miR-142-3p	1.28E-01	0.56	0.04	−0.51	0.70
hsa-miR-212	1.29E-01	−0.10	−0.53	−0.44	0.64
hsa-miR-518d-5p	1.30E-01	−0.12	0.08	0.19	1.14
hsa-miR-433	1.32E-01	0.03	−0.14	−0.18	0.89
hsa-miR-200b^*^	1.34E-01	−0.16	0.20	0.36	1.28
hsa-miR-491-5p	1.34E-01	0.02	−0.31	−0.32	0.80
hsa-miR-24	1.35E-01	0.16	−0.39	−0.54	0.69
hsa-miR-193b^*^	1.38E-01	−0.26	0.19	0.46	1.37
hsa-miR-130b^*^	1.38E-01	−0.13	0.18	0.31	1.24
hsa-miR-30c	1.40E-01	0.14	−0.22	−0.36	0.78
hsa-miR-23b	1.41E-01	0.13	−0.53	−0.66	0.63
hsa-miR-106b	1.42E-01	0.30	−0.32	−0.62	0.65
hsa-let-7b	1.42E-01	0.30	−0.14	−0.45	0.73
hsa-let-7d	1.43E-01	0.17	−0.43	−0.60	0.66
hsa-miR-647	1.43E-01	−0.29	0.04	0.33	1.26
hsa-miR-181a	1.43E-01	0.42	−0.95	−1.37	0.39
hsa-miR-130a	1.44E-01	0.16	−0.11	−0.28	0.82

* *Star strand of miRNA*.

**Table 3:** **qPCR analysis of select miRNAs in frontal cortex of NCI, aMCI, and AD subjects**.

**miRNA**	**aMCI fold Δ vs. NCI**	**AD fold Δ vs. NCI**
hsa-miR-668	0.87	0.85
hsa-miR-135b	0.76	0.84
hsa-miR-498	1.27	1.39^*^
hsa-miR-886-3p	0.85	0.51^**^
hsa-miR-132	0.58^**^	0.42^**^
hsa-miR-320a	0.98	0.91
hsa-miR-1252	1.17	1.14
hsa-miR-1273	1.03	1.22
hsa-miR-21	0.78	0.65^*^
hsa-miR-23a	0.53^**^	0.41^**^
hsa-miR-181b	0.88	0.93
hsa-miR-1827	1.27	1.21
hsa-miR-140-3p	0.85	0.62^*^
hsa-miR-33a	0.76	0.81
hsa-miR-150	1.43^*^	1.56^**^
hsa-miR-212	0.47^**^	0.41^**^
hsa-miR-23b	0.71^*^	0.62^**^
hsa-miR-106b	1.05	0.97
hsa-let-7d	0.73	0.66^*^
hsa-miR-181a	0.69	0.62^*^

* *p < 0.05*,

** *p < 0.01*.

**Table 4:** **qPCR analysis of select miRNAs and sirt1 mRNA in temporal cortex of NCI, aMCI, and AD subjects**.

**Probe set**	**aMCI fold Δ vs. NCI**	**AD fold Δ vs. NCI**
hsa-miR-212	0.96	0.63^*^
hsa-miR-132	0.88	0.65^*^
hsa-miR-23a	0.92	0.87
hsa-miR-23b	1.03	0.98
hsa-miR-150	1.32	1.47^**^
has-miR-668	0.95	1.01
sirt 1 mRNA	1.12	0.94

* *p < 0.05*,

** *p < 0.01*.

### Localization of miR-23a and sirt1 in the frontal cortex

**Figure 3:**
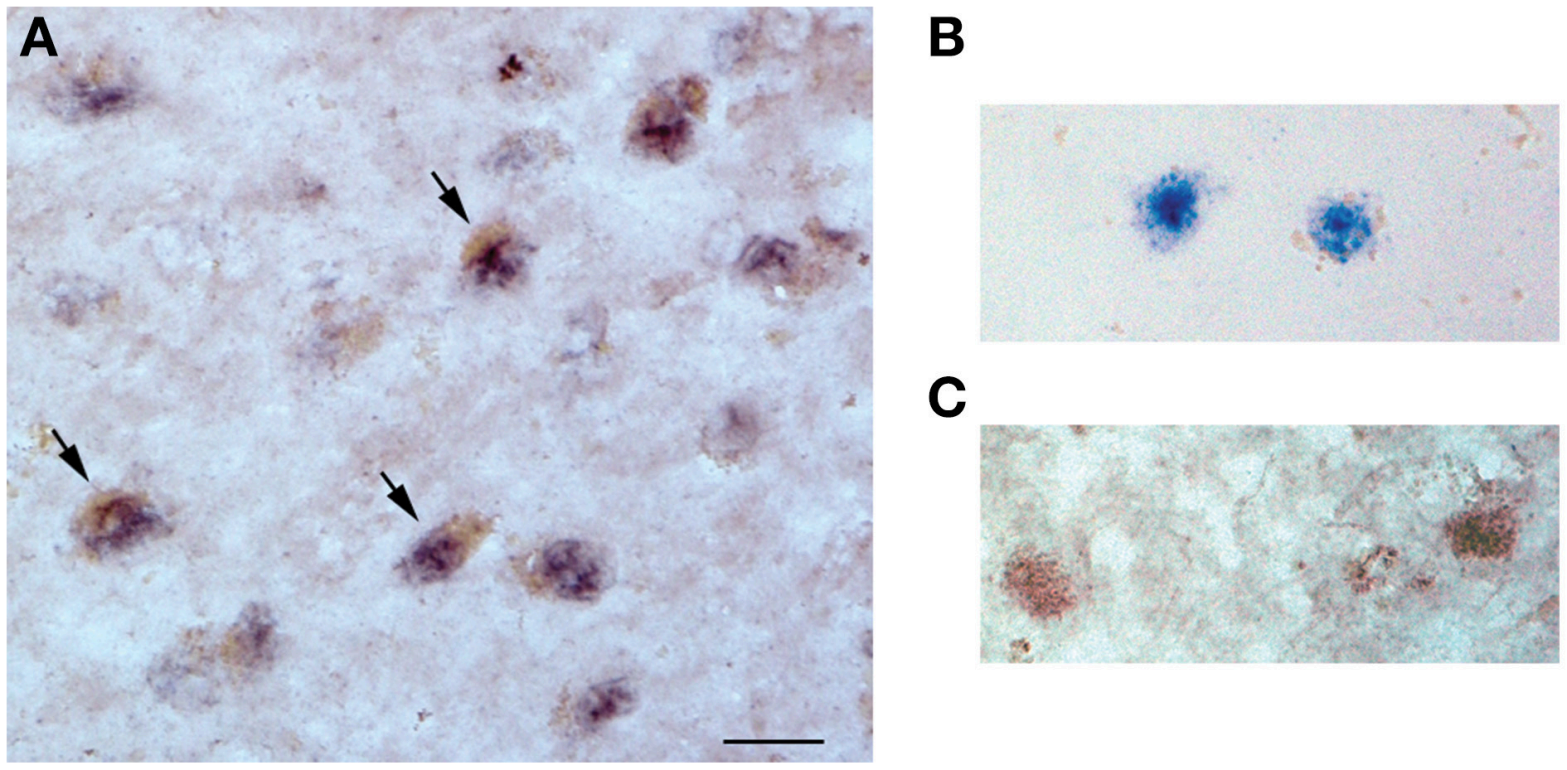
**miR-23a and sirt1 co-localize in the nucleus of frontal cortex layer III neurons**. Dual *in situ* hybridization/immunohistochemistry of miR-23a RNA and sirt1 protein expression was performed in frontal cortex of an 87 year-old female subject who died with a clinical diagnosis of aMCI. **(A)** Photomicrograph shows layer III neurons co-labeled with miR-23a labeling (dark purple) and sirt1 (brown, arrows). Note that miR-23a appears localized to the nucleus, whereas sirt1 labeling is nuclear but also with immunoreactivity in the cytoplasm. **(B)** Dual miR-23a/sirt1 detection in the absence of sirt1 primary antibody (anti-rabbit IgG only). **(C)** Dual miR-23a/sirt1 detection with control scrambled miRNA probe. Scale bar = 30 μm.

Dual miR-23a *in situ* hybridization and sirt1 immunohistochemistry revealed nuclear miR-23a labeling in frontal cortex layer III neurons in tissue obtained from an 87 year-old female subject who died with a clinical diagnosis of aMCI (Figure 3). Sirt1 protein immunoreactivity was found primarily in the nucleus but also within the cytoplasm of the same neurons, providing evidence that miR-23a has direct access to its sirt1 target and that miR-directed sirt1 regulation in cortex is neuronal in origin.

### miR-212 and miR-23a down-regulation increases sirt1 protein expression in human neuronal cells

**Figure 4:**
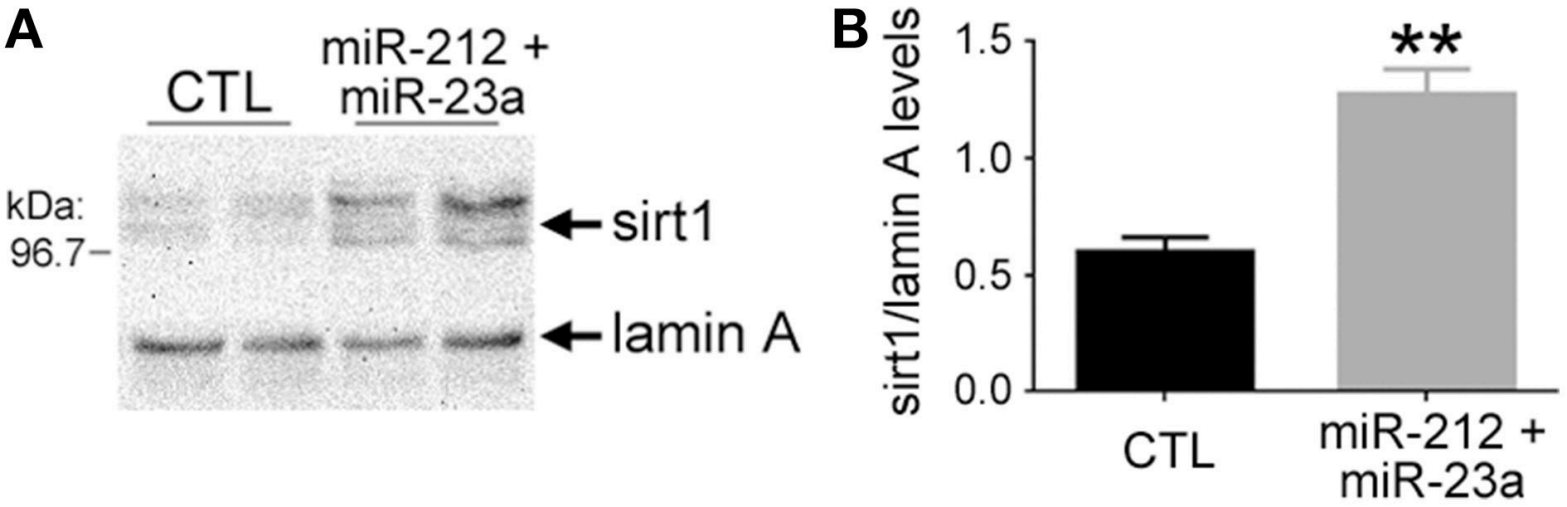
**Experimental down-regulation of miR-212 and miR-23a increases sirt1 protein expression**. Human hNT neuronotypic cells were co-transfected with small inhibitors of miR-212 and 23a or scrambled negative controls (CTL). Given the co-localization of miR-23a and sirt1 in the nucleus, cells were harvested 36 h following transfection with the miRNA inhibitors or controls and nuclear fractions were immunoblotted for detection of sirt1. **(A)** A representative western shows that miR-212/23a co-repression resulted in an increase in sirt1 immunoreactivity. **(B)** Quantitative analysis revealed that sirt1 levels were significantly increased by ~100% in miR-212/23a inhibitor-transfected cells compared to CTL-transfected cells. *n* = 6/group; **p < 0.01 via Student's unpaired test (two-tailed).

To determine the extent to which concomitant down-regulation of miR-212/132 and miR-23a/b and up-regulation of sirt1 observed in aMCI frontal cortex represented a functionally significant relationship, we treated human hNT neuronotypic cells with specific inhibitors of these miRNAs and measured sirt1 protein expression. Interestingly, inhibition of miR-212, miR-132, miR-23a, or miR-23b individually had no effect on sirt1 expression, but we found that concurrent inhibition of miR-212 and miR-23a resulted in a significant ~100% increase in sirt1 (Figure 4), whereas co-inhibition of miR-132 and miR-23a resulted in a ~40% increase in sirt1 (*p* < 0.05, data not shown). By contrast, miR-23b co-inhibition with miR-212 or miR-132 had no effect on sirt1 expression (data not shown).

### miR-23a and miR-212 down-regulation protects against Aβ_1−42_ induced cell death via sirt1

**Figure 5:**
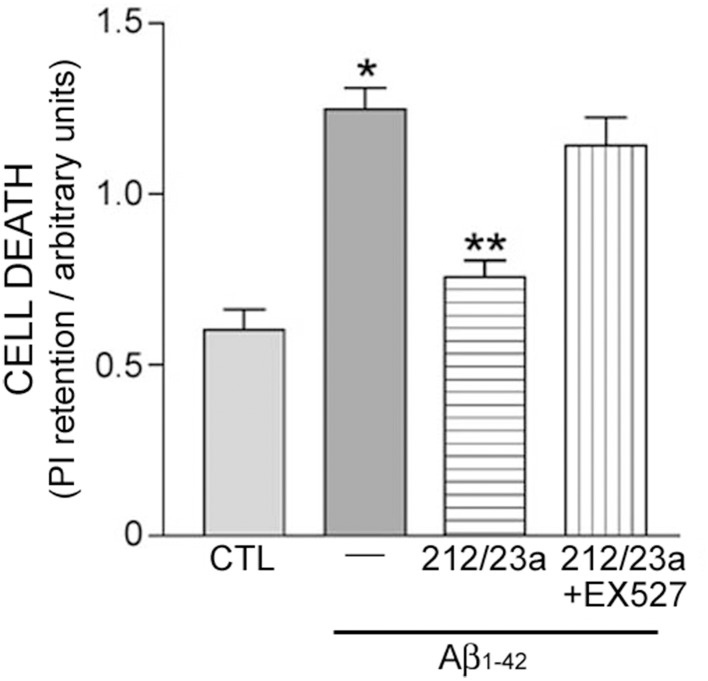
**Experimental down-regulation of miR-212 and miR-23a protects against Aβ_1−42_ in a sirt1-dependent manner**. Human hNT neuronotypic cells were co-transfected with small inhibitors of miR-212 and 23a or scrambled negative controls (CTL). Cells were then treated with 10 μM Aβ_1−42_ for 48 h in the presence or absence of 100 nM EX527, a sirt1-specific inhibitor, followed by incubation with propidium iodide (PI) as an assay for cell death. Bar graph shows that Aβ_1−42_ treatment resulted in significant cell death that was significantly reduced by miR-212/23a co-repression. This rescue effect was reversed by EX527, suggesting that the protective effects of miR-212/23a co-repression was mediated by sirt1. *n* = 8/group; *p < 0.05 vs. CTL; **p < 0.05 vs. Aβ_1−42_ via One-way ANOVA with Bonferroni correction for multiple comparisons.

To test whether miR-212 and miR-23a regulation of sirt1 results in neuronal protection, hNT cells were treated with inhibitors of either miRNA independently, or with inhibitors of both miRNAs combined, followed by challenge with Aβ_1−42_, which has been shown to induce cell death in these neuronal cells (Counts and Mufson, 2010). Inhibition of both miRNAs was sufficient to reduce Aβ_1−42_ induced cell death (Figure 5). This response was blocked by co-administration with the sirt1 inhibitor EX527 (Figure 5), supporting the concept that miRNA regulation of sirt1 expression is a viable neuroprotective pathway.

## Discussion

The discovery of miRNAs (Lagos-Quintana et al., 2001) introduced a new layer of complexity to gene regulation, but also afforded the opportunity to better understand the molecular underpinnings of cellular function and dysfunction. This concept has already been well-demonstrated in cancer research and the potential therapeutic value of targeting miRNAs has been gaining acceptance in that field (Ma and Weinberg, 2008). With respect to neurodegenerative disorders such as AD, the presence of miRNAs in the human brain and evidence for miRNA function in a wide variety of complex neuronal processes, including synaptic plasticity, suggests that miRNA regulation could have immense implications not only for disease pathogenesis but also for neuronal responses to progressive neurodegeneration (Kosik, 2006). Prior studies show that miRNAs could impact AD progression through several mechanisms. For instance, postmortem human tissue studies in well-characterized cohorts have shown decreased neocortical levels of miR-29a/b, miR-9, and miR-107 in AD compared to control subjects, which was associated with increased BACE1 mRNA expression and Aβ generation (Hébert et al., 2008; Wang et al., 2008; Che et al., 2014); in particular, miR-107 is downregulated very early in the disease process (Wang et al., 2008). By contrast, miR-15a is decreased in AD brain compared to healthy controls and is implicated in tau hyperphosphorylation *in vivo* (Hébert et al., 2010). Here, we show that mir-132/212 and miR-23a/b are selectively down-regulated in the frontal cortex in subjects clinically diagnosed with aMCI and that these alterations appear to be functionally linked to an up-regulation of sirt-1 and sirt-1 mediated protective responses. This novel finding adds to a growing literature on miRNA involvement in AD pathophysiology. However, rather than implicating another group of miRNAs in promoting neurodegeneration, our data support the concept that innate neuronal compensatory miRNA-mediated pathways are also activated in aMCI. A greater understanding of these and other miRNA pathways functioning during these putative prodromal stages of AD holds the promise that these pathways could be harnessed pharmacologically for drug development.

The miR-132/212 cluster has been implicated in several neuronal pathways, including dendritic elaboration (Magill et al., 2010) and learning and memory (Wang et al., 2013), and is downregulated in AD neocortex (Cogswell et al., 2008; Hébert et al., 2013; Lau et al., 2013; Wong et al., 2013). In particular, Wong and colleagues have previously shown that the miR-212/132 cluster is down-regulated in temporal cortex in AD, and that inhibition of miR-212 and/or miR-132 expression can induce apoptosis in primary neurons after 1 week in culture via activation of a foxo3a-mediated cell death pathway (Wong et al., 2013). In this regard, we replicated the finding that the miR-212/132 cluster is down-regulated in the temporal cortex in AD. However, we found no reductions in miR-212/132 in this region in aMCI; miR-212/132 was down-regulated in aMCI only in frontal cortex. In addition, in our hands experimental inhibition of miR-212 and/or miR-132 had no effect on cell survival of hNT neurons after 48 h in the absence of Aβ_1−42_. However, co-inhibition of either miR-212 or miR-132 with miR-23a conferred neuroprotection against Aβ_1−42_ in a sirt1-dependent manner. These discrepancies are likely explained by several factors. For instance, down-regulation of these miRNAs may have different functional implications in the AD temporal cortex, which displays a greater degree of degenerative changes than the aMCI frontal cortex. Notably, we found that sirt1 up-regulation in frontal cortex was confined to aMCI, suggesting that other AD-related pathways prevent sirt1 activation as the disease progresses. The down-regulation of miR-212/132 in aMCI frontal cortex may initially participate in protective compensatory mechanisms, but with sustained reductions these miRNAs may join a pathological cascade that promotes disease progression through foxo3a. In this regard, sirt1 activity has been shown to protect against foxo3a-mediated pro-apoptotic pathways by the deacetylation of this transcription factor (Brunet et al., 2004; Qin et al., 2008). Moreover, the differential roles for this cluster may depend on target binding partners, since miR-212/132 and miR-23a co-inhibition was required for sirt1 activation and neuroprotection. Hence, the activity of the miR-212/132 cluster may be context dependent during the progression of AD.

miR-23a/b has also been shown to be dysregulated in the AD brain (Cogswell et al., 2008; Lau et al., 2013), yet much less is known about this miRNA cluster in neuronal function. However, like miR-212/132, miR-23a levels have been inversely linked to apoptosis. In a recent report detailing mechanisms for neuronal cell death in a model of traumatic brain injury, *in vitro* studies revealed that miR-23a inhibition increased etoposide-induced cell death after 24 h in cortical neurons via caspase activation (Sabirzhanov et al., 2014). By contrast, we found that miR-23a inhibition alone had no effect on Aβ-induced cell death after 48 h, yet was neuroprotective in the presence of miR-212 by activating sirt1. Again, these discordances are not incompatible, but suggest that miR-23a has both pro-apoptotic and neuroprotective properties that depend on specific miRNA binding partners and whether these miRNAs are targeting pro-apoptotic factors such as caspases or, as presently shown, factors such as sirt1 that promote neuronal viability.

While the functional consequences of sirt1 up-regulation in this paradigm are unclear, sirt1 deacetylase activity plays an important role in regulating diverse cellular processes including aging, inflammation, and stress resistance (Imai et al., 2000; Brunet et al., 2004; North and Verdin, 2004; Herskovits and Guarente, 2014). In the adult brain, sirt1 can also modulate dendritic (Codocedo et al., 2012) and synaptic plasticity as well as memory formation (Gao et al., 2010; Michán et al., 2010). In addition to its importance during normal brain aging, sirt1 may also confer protective properties in neurodegenerative disorders such as AD (Qin et al., 2006, 2008; Min et al., 2010). Experimentally, sirt1 can reduce both Aβ-induced toxicity in neuronal cell lines (Conte et al., 2003; Han et al., 2004) and amyloid plaque formation in AD transgenic mice (Karuppagounder et al., 2009; Vingtdeux et al., 2010). Neuronal sirt1 expression has also been linked to non-amyloidogenic APP processing (Qin et al., 2006). Hence, we validated these concepts by showing that miR212/132 and miR-23a-mediated neuroprotection against Aβ is prevented by a sirt1-specific inhibitor. Sirt1 has also been shown to ameliorate tangle-like pathology in tau mutant mice (Kim et al., 2007; Min et al., 2010), possibly by tau deacetylation that allows ubiquitin ligases to target tau for degradation (Min et al., 2010; Cohen et al., 2011). Hence, there are several mechanisms by which miR-212/132 and miR-23a co-regulation of sirt1 expression may promote neuroprotection in the frontal cortex in aMCI. However, it is sirt1's role in the regulation and maintenance of dendritic growth (Codocedo et al., 2012) that could also play an important role in frontal cortex plasticity responses in MCI. In this regard, sirt1 knockout (KO) mice display reduced dendritic profiles relative to wild-type mice (Michán et al., 2010). Furthermore, overexpression of sirt1 can increase dendritic arborization, a phenotype that renders neurons resistant to dendritic dystrophy induced by Aβ (Codocedo et al., 2012). These data, taken together with multiple lines of evidence for a paradoxical up-regulation of synaptic elements within the frontal cortex during MCI (Counts et al., 2006; Bell et al., 2007; Williams et al., 2009; Bossers et al., 2010), suggest that sirt1 may mediate a synaptic remodeling response to mounting pathology during the progression of AD.

In summary, our data suggest that the transition from normal cognitive function in aging to a clinical diagnosis of aMCI may involve the suppression of brain microRNA networks such as miR212/132 and miR-23a. In the frontal cortex, this may result in the induction of cellular survival pathways including the stabilization of sirt1. Ultimately, as aMCI progresses to AD, the robust sirt1 expression is lost in frontal cortex, mimicking the situation seen in the temporal cortex. Recent microarray data suggest that the pattern of expression seen in sirt1 is also seen in other genes, particularly those involved in metabolic and synaptic machinery (Berchtold et al., 2013; Counts et al., 2013). Future research is necessary to confirm these findings in other AD cohorts, clarify whether the cumulative effect of these expression patterns is protective *in vivo* and by which sirt1-mediated mechanism, and determine whether augmentation of cell survival pathways such as sirt1 represents a viable strategy to delay neurodegeneration in prodromal AD. Linking these concepts to recent advances in miRNA knockdown and mimetic technology will usher in a new class of powerful *in vitro* and *in vivo* approaches to test the efficacy of miRNA-based therapies in preclinical AD models.

## Author contributions

RW participated in the experimentation and manuscript preparation. EM participated in the case selection, data analysis, and manuscript preparation. SC participated in the study design, experimentation, data analysis, and manuscript preparation.

### Conflict of interest statement

The authors declare that the research was conducted in the absence of any commercial or financial relationships that could be construed as a potential conflict of interest.
